# Scalable Synthesis of Mesoporous TiO_2_ for Environmental Photocatalytic Applications

**DOI:** 10.3390/ma12111853

**Published:** 2019-06-07

**Authors:** Francesca Petronella, Alessandra Truppi, Massimo Dell’Edera, Angela Agostiano, M. Lucia Curri, Roberto Comparelli

**Affiliations:** 1 CNR-IPCF, Istituto Per i Processi Chimici e Fisici, U.O.S. Bari, c/o Dip. Chimica Via Orabona 4, 70126 Bari, Italy; f.petronella@ba.ipcf.cnr.it (F.P.); a.truppi@ba.ipcf.cnr.it (A.T.); m.delledera@ba.ipcf.cnr.it (M.D.’E.); angela.agostiano@uniba.it (A.A.); 2 Università degli Studi di Bari “A. Moro”, Dip. Chimica, Via Orabona 4, 70126 Bari, Italy

**Keywords:** photocatalysis, titanium dioxide, mesoporous, nanomaterials, environmental remediation, water remediation, NO_x_, VOCs

## Abstract

Increasing environmental concern, related to pollution and clean energy demand, have urged the development of new smart solutions profiting from nanotechnology, including the renowned nanomaterial-assisted photocatalytic degradation of pollutants. In this framework, increasing efforts are devoted to the development of TiO_2_-based nanomaterials with improved photocatalytic activity. A plethora of synthesis routes to obtain high quality TiO_2_-based nanomaterials is currently available. Nonetheless, large-scale production and the application of nanosized TiO_2_ is still hampered by technological issues and the high cost related to the capability to obtain TiO_2_ nanoparticles with high reaction yield and adequate morphological and structural control. The present review aims at providing a selection of synthetic approaches suitable for large-scale production of mesoporous TiO_2_-based photocatalysts due to its unique features including high specific surface area, improved ultraviolet (UV) radiation absorption, high density of surface hydroxyl groups, and significant ability for further surface functionalization The overviewed synthetic strategies have been selected and classified according to the following criteria (i) high reaction yield, (ii) reliable synthesis scale-up and (iii) adequate control over morphological, structural and textural features. Potential environmental applications of such nanostructures including water remediation and air purification are also discussed.

## 1. Introduction

In recent years, one of the most important concerns of the scientific community and society has been health and environmental protection via a smart and sustainable use of natural resources. In this context, water resources are gaining increasing attention due to the occurrence of emerging pollutants including dyes, pharmaceutical and personal care products, endocrine disruptors, pathogens [1,2]. Moreover, the increasing amount of atmospheric pollutants has been regarded among the main causes of respiratory diseases such as emphysema, and bronchitis arising from the contact of NO_x_ with lungs [3].

Unfortunately, conventional pollution remediation methods show limited performances. For instance, in the field of water treatment adsorption or coagulation methods aim at concentrating pollutants by transferring them to other phases; sedimentation, filtration, chemical and membrane technologies involve high operating costs and can generate toxic secondary pollutants in the ecosystem [4]; and chlorination, although widely used in disinfection processes, can generate by-products associated with cancer or other pathologies [5].

It turns out that the interest of the scientific community has been focusing on alternative methods such as the “advanced oxidation processes (AOPs)”. AOPs are convenient innovative alternatives to conventional wastewater treatment processes [6,7] because they include a set of water treatment strategies such as ultraviolet (UV), UV-H_2_O_2_ and UV-O_3_, and semiconductor-based photocatalysis that aim at accomplishing the complete mineralization of organic pollutants (i.e., their conversion into safe by-products such as O_2_, H_2_O, N_2_ and mineral acids). Among AOPs, TiO_2_-based photocatalysis has recently emerged as a promising water treatment [8]. Photocatalysis takes place upon the activation of a semiconductor with electromagnetic radiation from sun or artificial light. When exposed to electromagnetic radiation, a semiconductor absorbs photons with sufficient energy to inject electrons from the valence band (VB) to its conduction band (CB), generating electron hole pairs (e^−^/h^+^). The h^+^ have an electrochemical potential sufficiently positive to generate ^•^OH·radicals from water molecules adsorbed onto the semiconductor surface, while the e^−^ react with oxygen molecules to form the superoxide anions, ^•^O_2_^−^, that quickly react with H^+^ to finally produce ^•^OH radicals after a series of concatenated reactions [9,10]. The overall photocatalytic efficiency depends on (i) the competition between e^−^/h^+^ recombination events and generation of reactive oxygen species (ROS) (ii) the competition between e^−^/h^+^ recombination events and e^−^/h^+^ trapping on semiconductor surface.

In this respect, TiO_2_ nanoparticles (NPs) are extremely advantageous due to their high photoactivity, high chemical and photochemical stability, high oxidative efficiency, non-toxicity and low cost. In addition, the size-dependent band gap of nanosized semiconductors allows tuning the e^−^ and h^+^ red-ox potentials to achieve selective photochemical reactions [11,12,13]. 

Remarkably, the reduced dimensions of TiO_2_ NPs imply a high surface to volume ratio, which ensures a high amount of surface-active sites even upon immobilization of the photocatalyst onto substrates, thus avoiding the typical drop in performance due to the immobilization of bulk TiO_2_. Immobilization is an essential requirement for a real application of TiO_2_ NPs, both for safety and technological reasons [6]. Indeed, immobilization may limit accidental release of nanomaterials, thus preventing TiO_2_ NPs turning into a secondary pollution source, and, at the same time, enables recovery and reuse of the photocatalyst. In fact, NPs have been demonstrated to harmfully impact on ecosystems, as reported in recent studies that have also shown that both TiO_2_ NPs and TiO_2_ NPs aggregates, at concentration higher than 10 mg/L, provoke hatching inhibition and malformations in the embryonic development of a model marine organism [14]. 

A great deal of work has been focused on improving the photoactivity of TiO_2_ NPs and extending its optical response in the visible light range. Indeed, excellent reviews [15,16,17,18,19] and original papers [11,20,21,22,23] have overviewed the huge number of synthesis strategies aimed at purposely tailoring TiO_2_ NPs by surface modification, doping, introduction of a co-catalyst, and crystalline structure manipulation. 

Among the numerous strategies devoted to properly designing the morphological complexity of TiO_2_ NPs, the possibility of obtaining mesoporous TiO_2_ is attracting increasing interest [24]. The International Union of Pure and Applied Chemistry (IUPAC) classifies porous solids in three groups according to their pore diameter: namely microporous (diameter not exceeding 2 nm) and mesoporous (diameter in the range from 2 nm to 50 nm) and macroporous (diameter exceeding 50 nm) [25]. The porosity arises from the ordered or disordered assembly of individual nanocrystals (NCs) in larger structures (mesostructures). Ordered structures result from a regular arrangement of pores in the space and show a narrow pore size distribution, conversely disordered structures are characterized by a random aggregation of NPs, that gives rise to a large pore size distribution [23]. 

As a result, TiO_2_-based mesoporous materials combine the well-known photocatalytic activity of TiO_2_ with peculiar textural properties, including pore sizes and high specific surface areas, typical of NPs. Such features may contribute to increase the amount of absorbed organic pollutants and to dissolve the O_2_ that can get to the TiO_2_ surface thus improving the efficiency of the mineralization process [24]. Mesoporous TiO_2_ NPs are regarded as promising adsorbents for various pollutants in water [26], as they present a high concentration of hydroxyl groups (−OH) on the surface, that allows adsorption of water pollutants and improves ^•^OH radicals’ generation, resulting in also being prone to further functionalization. 

Moreover, TiO_2_-based mesostructures and superstructures, such as hollow spheres, mesoporous TiO_2_ nanotubes and mesoporous TiO_2_ microspheres, enable multiple diffractions and reflections of incident UV light within the inner cavities, thus favoring a more efficient photogeneration of e^−^/h^+^ pairs, resulting in an improvement of the photocatalytic activity [27,28,29].

The present review aims at describing selected protocols, among the most interesting ones recently reported, for the synthesis of mesoporous TiO_2_ with advantageous properties in terms of size/shape distribution, crystallinity and textural characteristics. Specifically, the presented synthesis protocols have been identified as suited to be implemented for a large-scale TiO_2_ production, being scalable, cost-effective and relying on the use of safe chemicals. The high interest in the large scale manufacturing of nanoscale TiO_2_ can clearly be seen when looking at the expectation of the complete conversion of TiO_2_ production from bulk to nanomaterialt is foreseen to occur by 2025 with a production close to 2.5 million metric tons per year [30]. The review is mainly focused on sol-gel techniques and hydrothermal routes, namely soft templating approaches that make use of removable structure-directing agents as surfactant micelles, block copolymers, ionic liquids and biomacromolecules. All the reported protocols are suited for a viable scale-up because they make use of water as reaction solvent, and match the requirements of low-cost precursors, relatively low synthesis temperatures and high reaction yield.

Finally, an overview of the latest environmental applications of TiO_2_ for water remediation and air purification will be presented.

## 2. Synthesis of Mesoporous TiO_2_

### 2.1. Sol-Gel Methods

The sol-gel approaches [31] are among the most investigated techniques applied to obtaining ceramic or glass materials, having the advantages of being reproducible, industrially scalable and highly controllable.

The soft template processes underlying sol-gel strategies are generally based on several steps: (i) preparation of the solution of a selected TiO_2_ precursor; (ii) hydrolysis of TiO_2_ precursor in the presence of a suitable surfactant; (iii) removal of the solvent in order to facilitate the generation of the gel; (iv) condensation reaction; and (v) calcination for the complete removal of surfactant, solvent and unreacted precursor.

**Figure 1:**
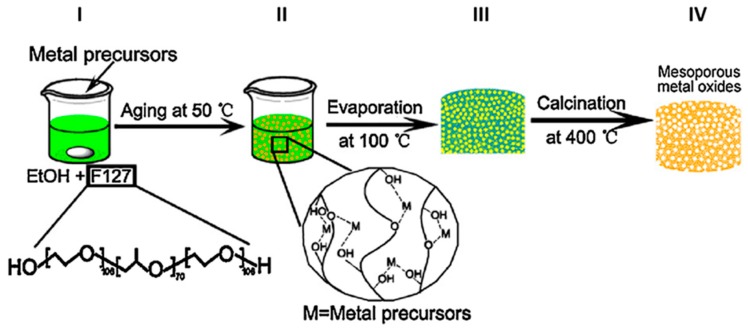
General synthetic scheme for the production of mesoporous metal oxides according to the evaporation-induced self-assembly method (EISA). The first step consists in the preparation of an ethanol solution containing the metal precursor (Ti(OBu)_4_ for TiO_2_) and the Pluronic F127 as templating agent (**I**). The mixture is kept at 50 °C for 24 h in order to induce the coordination bonds between the metal ions (M) and oxygen-containing group of F 127 (**II**). The subsequent thermal treatment at 100 °C for 6 h (**III**) promotes the formation of a xerogel of the metal-F127 hybrids. The final calcination at 400 °C (**IV**) is intended to remove of organic molecule and results in the formation of mesoporous metal oxides. Reprinted with the permission of ref. [33]. Copyright © 2019 4542370045937.

Among the sol-gel synthetic approaches the EISA (evaporation-induced self-assembly, Figure 1) has been recently applied for the preparation of metal oxides including TiO_2_. The main feature of the EISA method is the use of a surfactant as a templating agent. Triblock copolymers as P123 (Poly(ethylene glycol)-block-poly(propylene glycol)-block-poly(ethylene glycol)) and F127 (poly(ethylene oxide) poly(propylene oxide)-poly(ethylene oxide)), are recognized as the most promising surfactants used for this method [32]. Indeed, surfactant selection represents one of the most critical parameters of EISA approaches because its chemical and physical properties affect the textural properties of the resulting material that can be deposited as a thin film on a suitable substrate. 

A typical sol-gel EISA synthesis of TiO_2_ starts with the preparation of a solution containing Pluronic F127 in absolute alcohol (EtOH), and the subsequent addition of titanium butoxide Ti(OBu)_4_ under vigorous stirring (Figure 1, I). The resulting suspension is kept at 50 °C for 24 h, and then dried at 100 °C for 6 h (Figure 1, II and III respectively). The as-prepared product shows a texture compatible with xerogels. The final calcination at 400 °C is carried out at specific heating rate in order to induce the removal the block copolymer surfactant species (Figure 1, IV). At this stage, aggregates formed by NPs of 5–10 nm in size have been produced, thus resulting in a mesoporous product with a specific surface area of 145.59 m^2^/g and an average pore size of 9.16 nm [33]. 

M.G. Antoniou et al. reported a similar approach to obtain a mesoporous TiO_2_-based coating for photocatalytic applications. The TiO_2_ sol, comprised of titanium tetraisopropoxide (TTIP), acetic acid, isopropanol and Tween 80 as surfactant, is applied by dip-coating on glass substrate and then it is heated at 500 °C to remove the surfactant template. The dip-coating–calcination cycle is repeated 3 times for each deposition, resulting in uniform and transparent mesoporous nanocrystalline TiO_2_ films with high surface area (147 m^2^/g), porosity (46%) and anatase crystallite size of 9.2 nm. The amount of photocatalyst per cm^2^ is estimated to be 62.2 mg/cm^2^ with an overall coated area, considering both sides of the substrate, of 22.5 cm^2^ [34].

An alternative strategy has been proposed to further increase the specific surface area of mesoporous TiO_2_, that indicates the use of two types of TiO_2_ precursors such as TiCl_4_ and TTIP in a suitable molar ratio, with TiCl_4_ playing the two-fold role of precursor and pH stabilizer. A solution containing a defined TiCl_4_:TTIP:P123:ethanol ratio is stirred for 3 h at room temperature and the resulting product is suitable to be deposited by spin coating on glass substrates. After drying at room temperature for 24 h, the samples is thermally treated at 130 °C for 2 h to promote crossing-linking and prevent possible cracks in the film and collapsing of the mesostructure due to the high temperature. The final calcination treatment is carried out by heating stepwise up to 400 °C [35]. 

**Figure 2:**
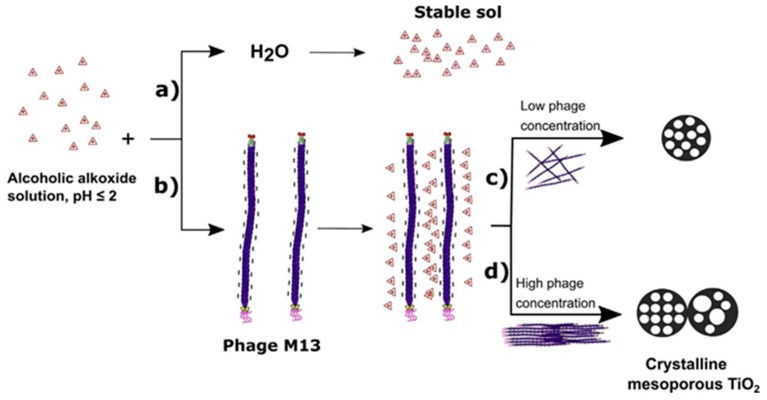
Proposed mechanism of mesoporous TiO_2_ synthesis: consist in the preparation of a titanum alkoxide (titanium(tetra)isopropoxide) solution at pH ≤ 2. A vary stable sol is obtained with acid aqueous solution (pH 1–2) (**a**); the sol-gel reaction is performed with Phage M13 and a well-established structure is obtained (**b**). A local order of pores and macropores can be obtained at high phage concentration (**d**), while disordered pores with a narrow pore size distribution at a low concentration (**c**). Reproduced with permission from [36]. Copyright © 2019 4541961016973.

A recently reported sol-gel synthetic approach for the production of TiO_2_ makes use of a biological template, namely the bacteriophage M13, a rod-shaped virus that is able to control the alkoxide condensation in the sol-gel process allowing the formation of mesopores having a diameter that can be tuned by adjusting only the reaction pH. Remarkably, the resulting product exhibits exceptional thermal stability of the anatase phase, which stays as the predominant phase even after a thermal treatment at 800 °C, that, in fact, promotes an increase in the pore and crystal size (Figure 2) [36].

One of the main goals in the synthesis of mesoporous TiO_2_ for environmental photocatalytic applications is to increase the TiO_2_ optical response in the range of visible light. For this purpose, synthetic approaches have been developed to accomplish this result. For instance, a mixture of polyethylene glycol (PEG) and polyacrylamide (PAM), has been used as the templating agent. PAM and PEG are slowly introduced in a mixture of deionized water, nitric acid (8%), ethanol and Ti(OBu)_4_ as TiO_2_ precursor. The resulting white gel is dried until a light-yellow powder is obtained that undergoes two calcination steps: the first in nitrogen atmosphere, and the second in air. In the first calcination step, three different temperature values are investigated: 500 °C, 600 °C and 700 °C respectively, while the second calcination step is carried out at 500 °C. The authors have demonstrated how increasing PAM mass the gel formation rate increases, due to the improved interaction between amide groups of PAM with the hydroxyl groups of the TiO_2_ sol. The PEG prevents the mesostructure collapsing during the first thermal treatment. Moreover, the molecular weight (MW) of PEG has been reported to increase as the crystallite size increases and the specific surface area decreases. The obtained mesoporous TiO_2_ is found to have a specific surface area measured by BET (Brunauer–Emmett–Teller) test between 104.25 and 110.73 m^2^/g and a pore size (measured by Barret-Joyner-Halenda isotherm) between 16.92–16.80 nm; being the variation of the specific surface area and pore size values affected by the variation of the molecular weight of the PEG used in the synthesis. The authors point out that the two calcination steps improve the textural properties of the TiO_2_ because they promote a higher crystallinity, and allow to achieve a homogenous porosity, and a higher specific surface area. In particular, the first calcination step under N_2_ atmosphere causes the conversion of PEG (less thermally stable than PAM) in amorphous carbon, which plays the role of a scaffold around pores, thus preventing the mesostructure from collapsing [37]. Furthermore, the small amount of amorphous carbon is able to induce a doping effect, and therefore the obtained photocatalyst is able to extend its photoactivity to visible range, as demonstrated in the ultraviolet–visible (UV–Vis) reflectance spectrum, that shows an increase, in the visible range, of the Kubelka–Much function intensity. 

Also, Phattepur et al. have synthesized mesoporous TiO_2_ with an innovative sol-gel technique by using lauryl lactyl lactate as biodegradable and inexpensive additive to control the size of large inorganic cluster. The nanostructured photocatalyst is prepared by using Ti(OBu)_4_ as precursor in a solution containing a defined amount of lauryl lactyl lactate (0.25 mL, 0.5 mL, 0.75 mL and 1 mL), ethanol, and hydro-chloric acid. Such a solution is mixed with a second solution of ethanol and distilled water under vigorous stirring for 8 h up to the generation of the gel. The mixture is, then, aged, dried, ground and finally calcined, resulting in a final product with a specific surface area up to 40.10 m^2^/g, a pore volume 0.112 cm^3^/g [38].

The interest towards colloidal routes for the synthesis of mesoporous TiO_2_ is further supported by a recently granted US patent [39]. The synthetic scheme consists of an acid-catalyzed hydrolysis of a water soluble TiO_2_ precursor as TiOCl_2_ or TiOSO_4_, occurring in the presence of a porogen molecule, namely an organic alpha hydroxyl carboxylic acid, as citric acid, at relatively low temperatures (up to 100 °C). Such a procedure allows 100 nm spherical mesoporous anatase NPs to be achieved and control of the pore size by varying the molar ratio between the TiO_2_ precursor and the organic alpha hydroxyl acid. Remarkably, these mesoporous TiO_2_ NPs show a bimodal pore size distribution. Such bimodal porosity is due to the presence of both intra-particle and inter-particle pores. Intra-particle pores (from 2 nm to 12 nm in size) are detected in individual TiO_2_ NPs while the inter-spatial fissures of dimensions from 15 nm to 80 nm, due to the packed arrangement of the NPs, result in inter-particle pores [39].

### 2.2. Synthesis in Room Temperature Ionic Liquids 

Room-temperature ionic liquids (RTILs) are regarded with increasing interest both in academia and industry [40]. In the synthesis of TiO_2_ NCs, the use of RTILs offers multifold advantages including the control over the morphology and phase composition, the colloidal stability [41,42] and the possibility to achieve a large scale production of photocatalytic TiO_2_ [41,43,44]. Indeed RTILs allow, in principle, to perform synthesis of titania at low temperature as they are organic salts characterized by a low melting point (lower than 100 °C), thus resulting in being liquid and thermally stable over a wide temperature range [45]. In a typical synthesis of TiO_2_ NPs, the 1-butyl-3-methylimidazoliumtetrafluoroborate (BF) is used as solvent and TiCl_4_ as TiO_2_ precursor. After mixing BF and TiCl_4_, purified water is added slowly under vigorous stirring at room temperature to promote the immediate hydrolysis of TiCl_4_ indicated by the appearance of turbidity. Such a turbid solution is stirred at 80 °C for another 12 h and the resulting product is collected by centrifugation upon dilution with water in order to decrease the viscosity due to the BF. The residual solvent is removed by extraction with acetonitrile, soluble to both the inorganic species and the RTIL, in a closed vessel at 50 °C for 8 h and the final product is dried in a vacuum oven at 40 °C [43]. Such a synthetic approach is used to synthesize mesoporous N-doped TiO_2_ (10–50 nm in size) in anatase phase, with 5–8 nm pores observed by transmission electron microscopy (TEM) and a band gap, determined by diffuse reflectance spectroscopy, of 2.47 eV [46].

### 2.3. Hydrothermal Synthetic Methods

A typical hydrothermal process is carried out in an autoclave, possibly equipped with Teflon liners and a closed system under controlled temperature and/or pressure (room temperature and at pressure >1 atm) [47]. The temperature can be higher than the boiling point of water corresponding to the pressure of vapor saturation [48].

Hydrothermal processes are extremely attractive for the large-scale production of mesoporous TiO_2_ NPs, because they (i) are environmentally friendly, (ii) make use of aqueous solutions, (iii) do not require any post-calcination treatment and (iv) allow a facile recovery of the photocatalyst after the synthesis [48,49,50].

TiO_2_ NPs prepared by hydrothermal methods show several advantages, including high crystallinity, reduced particle size, uniform size distribution, prompt dispersibility in polar and non-polar solvents and a stronger interfacial adsorption; moreover, they enable the easy fabrication of high-quality coatings on several supporting material [51].

In hydrothermal synthesis, temperature, filling volume [52] pressure [53] pH and treatment duration are regarded as the key parameters to control the resulting morphological and structural properties of TiO_2_. Under hydrothermal conditions, a decrease of the reaction temperature cause a particle size decrease and an increase of particle agglomeration [47]. The growth of TiO_2_ NPs is also possible by using a template-based technique, taking advantage of the use of suitable high-molecular-weight surfactants, able to promote structural self-assembly [47].

The hydrothermal method applied on TiO_2_–nH_2_O amorphous gel, considered as TiO_2_ NPs precursor, has been widely used to prepare nanocrystalline titania [54]. This treatment can be carried out either in pure distilled water or in the presence of mineralizing species, such as hydroxides, chlorides and fluorides of alkali metals at different pH values [54]. Kolen’ko et al. have developed the synthesis of ultrafine mesoporous titania powders in anatase phase via hydrothermal process starting from TiO_2_–nH_2_O amorphous gel. The preparation of TiO_2_–nH_2_O amorphous gel requires multiple steps starting from the high-temperature hydrolysis of complex titanyl oxalate acid (H_2_TiO(C_2_O_4_)_2_) aqueous solutions. Briefly, the preparation route of H_2_TiO(C_2_O_4_)_2_ aqueous solution is based on: (I) the preparation of H_2_TiCl_6_ from TiCl_4_ and chloride acid (HCl), (II) hydrolysis of H_2_TiCl_6_, (III) wash of TiO_2_–nH_2_O by distilled water, and (IV) dissolution of TiO_2_–nH_2_O in the oxalic acid. At this stage the TiO_2_–nH_2_O is treated in a polytetrafluoroethylene (PTFE)-lined autoclave at temperature of 150 °C or 250 °C for a period of time ranging from 10 min to 6 h. After the treatment in autoclave the sample is cooled down to room temperature, the obtained product is centrifuged, washed and dried at 80 °C [54]. Washing procedures are essential in order to collect the photocatalyst and remove the templating agent and salts that can possibly obstruct the pores, thus achieving the desired porosity and specific surface area [55,56]. The size of the mesoporous anatase particles can be controlled in the range of 60–100 nm by adjusting the concentration of H_2_TiO(C_2_O_4_)_2_ aqueous solution that is expected to affect the amount of TiO_2_ nuclei.

Hydrothermal synthesis allows also a fine control over NP morphology. Indeed, mesoporous TiO_2_ microparticles with a well-defined spherical shape in the range of 2–3 µm, and a crystallite size in the range from 7.3 nm to 22.3 nm have been prepared by hydrothermal reaction of poly(ethylene glycol)-poly(propylene glycol)-based triblock copolymer and TTIP mixed with 2,4-pentanedione [57]. The surfactant solution is prepared dissolving the triblock copolymer in distilled water at 40 °C and adding sulfuric acid. Successively, TTIP is mixed with 2,4-pentanedione and slowly added dropwise into the previously prepared surfactant solution. The reaction is carried out at 55 °C for 2 h without stirring and a light-yellow powder is obtained. The hydrothermal treatment is performed at 90 °C for 10 h followed by an annealing step, which is necessary to remove the residual surfactant. 

Zhou and co-workers have proposed titanium sulfate (Ti(SO_4_)_2_) as a precursor of TiO_2_ in the presence of urea to obtain microspheres by hydrothermal treatment TiO_2_ [58], by using reaction time as a key parameter to control average crystallite size, pore size and volume, and the specific surface area.

Mesoporous TiO_2_ anatase microspheres have been also successfully obtained with a simple one-step hydrothermal synthesis by Lee et al. [49]. The titanium-peroxo complex is treated with nitric acid, 2-propanol, NH_4_OH in Teflon-lined autoclave at 120 °C for 6 h. The final product is filtered, washed with distilled water several times until the pH reaches 7 and dried at 65 °C for 1 day, obtaining the mesoporous material without any post-calcination procedure. In particular, the size of mesoporous TiO_2_ anatase microspheres lays in a dimensional range between 0.5 µm and 1 µm. According to high-resolution TEM (HRTEM) analysis, the formation of a microsphere (secondary particle) results from the aggregation of diamond-shaped TiO_2_ NCs (50 nm × 20 nm) (primary NPs). The authors have suggested that during the hydrothermal reaction the aggregation of individual diamond-shaped TiO_2_ NCs in secondary particles, is thermodynamically favored with respect to the formation of primary TiO_2_ NPs. Interestingly, the porosity of the microspheres is ascribable to the interspaces between TiO_2_ NPs assembled to form a microsphere [49].

Similarly, Santhosh et al. have synthesized mesoporous TiO_2_ microspheres by a facile hydrothermal reaction carried out at 120 °C for 24 h that, instead, makes use Ti(OBu)_4_ as precursor [59]. The material structure is based on TiO_2_ having a diameter in a range from 100 nm to 300 nm. The corresponding specific surface area is 56.32 m^2^/g and the bimodal pore structure shows pore width of 7.1 nm and 9.3 nm respectively [59].

Hollow TiO_2_-based core-shell structures have been also reported and they are expected to show a high photocatalytic activity because their unique morphology allows the multiple reflection of UV light within the inner cavity [60]. Furthermore, they display as valuable advantages high surface-to-volume ratio, low density and low production cost. Recently, Cui et al. have reported a facile one-step hydrothermal method to synthetize a mesoporous hollow core-shell structured TiO_2_ microspheres. PEG (MW 2000) has been used as soft templating agent to obtain Ti^4+^-PEG globules suited for the generation of hollow structures. The control on crystallite size, microsphere size, shell thickness and the roughness are achieved by tuning the duration of the hydrothermal treatment. In particular, increasing the reaction time, the crystallite and microsphere size, the shell thickness and the roughness increase while the core size decreases [60].

**Figure 3:**
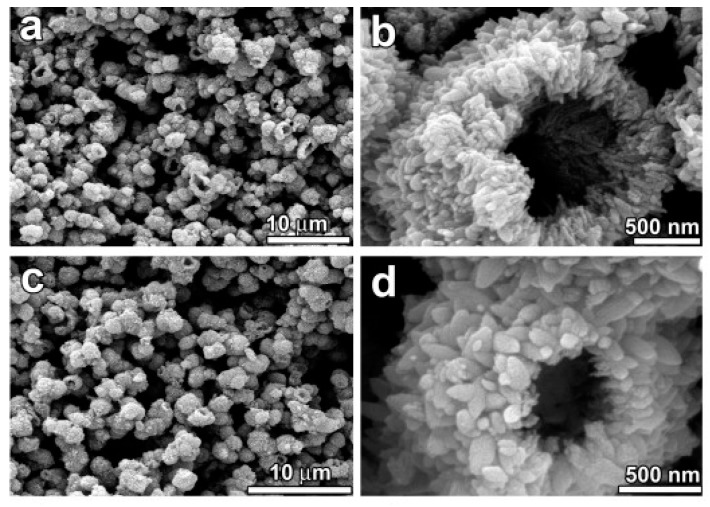
Large–scale synthesis of TiO_2_ hallow microspheres has been obtained by Ye et al. by using a hydrothermal treatment following the calcination. The morphology and microstructure of the hollow microspheres have been characterized by scanning electron microscopy (SEM) (**a**–**d**), in order to evaluate the effect of the calcination. (**a**,**b**) SEM micrographs of the not calcined sample: low-magnification and high-magnification, respectively. (**c**,**d**) SEM micrographs of the TiO_2_ hollow microspheres calcined at 500 °C for 2 h: low-magnification and high-magnification, respectively. SEM micrographs of the non-calcined material show that the sample consisting of large-scale hollow microspheres, being the shell of the microsphere itself made of numerous nanoparticles (NPs). Interestingly, the SEM micrographs of calcined material indicate an excellent thermal stability of the hallow microsphere. The morphology and the mean external diameter of the hollow microspheres has been found unchanged after the calcination treatment at 500 °C. Reproduced with permission from [61]. Copyright © 2018 Elsevier.

Ye et al. have developed an interesting strategy for the large–scale synthesis of hallow microspheres by performing a hydrothermal treatment followed by a calcination step (Figure 3) [61]. They have used potassium titanium oxalate (PTO) as Ti precursor performing the hydrothermal process in autoclave at 150 °C for 4 h, thus obtaining 1.75 µm microspheres composed of TiO_2_ NPs. In particular, a strong effect of calcination temperature on the photocatalytic performance of the photocatalyst is reported, with the hollow TiO_2_ microspheres calcined at 500 °C showing the highest photocatalytic activity. 

**Figure 4:**
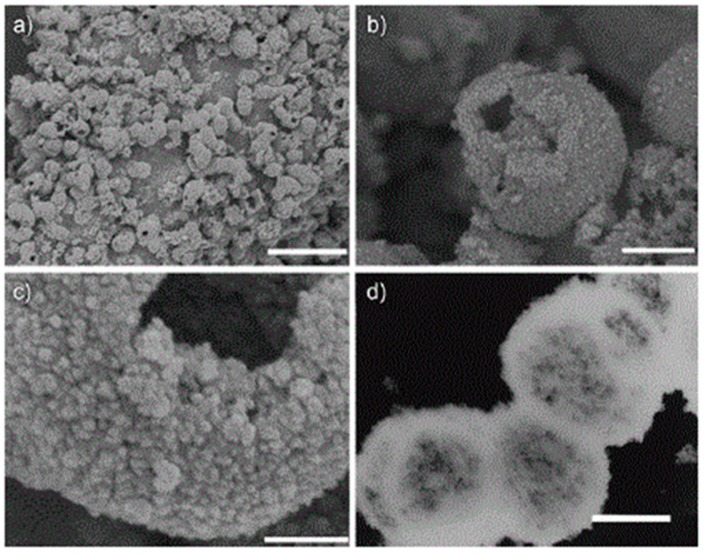
Large-scale synthesis of porous TiO_2_ hollow aggregates reported by Liu et al. The synthesis is carried out using a low-temperature hydrothermal method without templates. (**a**–**c**) Field-emission scanning electron microscopy (FESEM) analyses at different magnifications and (**d**) transmission electron microscope (TEM) micrograph of the “as prepared” sample by one-step hydrothermal treatment at 160 °C for 6 h. The scale bars for (**a**–**d**) are 5 mm, 500 nm, 100 nm, and 500 nm, respectively. The low-magnification FESEM micrograph (**a**) of the sample indicates that the aggregates are composed of a large amount of NPs. While, the micrograph (**b**) shows the hollow interior of the single aggregate and the micrograph (**c**) at a higher magnification highlights the porous structure of the TiO_2_ NP aggregates. Moreover, the hollow structure of the TiO_2_ sample is confirmed by TEM micrograph (**d**). Reproduced with permission from [62]. Copyright © 2018 John Wiley and Sons.

TiO_2_ hollow spheres aggregates, characterized by porous walls, can be also synthesized on large-scale by performing the hydrothermal hydrolysis of Ti(SO_4_)_2_ assisted by NH_4_F without employing any templates [62]. The resulting hollow material, reported in Figure 4, presents a high surface area, smaller crystal size, and highly porous structure [62] Several hydrothermal protocols have been also successfully developed to synthesize anisotropic mesoporous NPs. Anisotropic TiO_2_ NCs have been extensively investigated for several energy conversion applications, since their peculiar morphology enables a facile charge transport along the longitudinal dimension and decreases the e^−^/h^+^ recombination rate [51,52].

Mesoporous TiO_2_ nanotubes (TNTs) are materials of great interest due their high ion-exchange capability, relative stability, enhanced conductivity and high specific surface area [63,64]. In addition, TNTs contain a large amount of hydroxyl groups respect to spherical TiO_2_ and may find an effective use as ion adsorbent systems. Sattarfard et al. have prepared mesoporous TNTs by hydrothermal synthesis starting from TiO_2_–P25 NPs as titania precursor [63].

In this protocol, TiO_2_–P25 powder is introduced into NaOH aqueous solution and held at 115 °C for 24 h in a stainless-steel with Teflon lining reactor placed in an oil bath. Afterwards, the mixture is cooled to room temperature and centrifuged, and the obtained precipitate requires several washing, recovery and thermal treatment steps to finally obtain TNT with a specific surface area of 200.38 m^2^/g and an average outer and inner diameter approximately of 9 nm, 4 nm, respectively, with a wall thickness of 2.5 nm. Remarkably, the use of a strong aqueous base as NaOH as dispersing agent for TiO_2_ P25 and the subsequent hydrothermal treatment are the key steps that determine the formation of titanate tubular structures, while washing with HCl has been demonstrated to be essential to convert tubular titanate in TiO_2_ nanotubes. Indeed, the treatment of TiO_2_ NPs with NaOH is known to break Ti–O–Ti bonds, thus resulting in the formation of sheets, while the subsequent washing with acid or water, reducing the electrostatic charge, induces the folding of the sheets yielding the formation of nanotubes [63].

**Figure 5:**
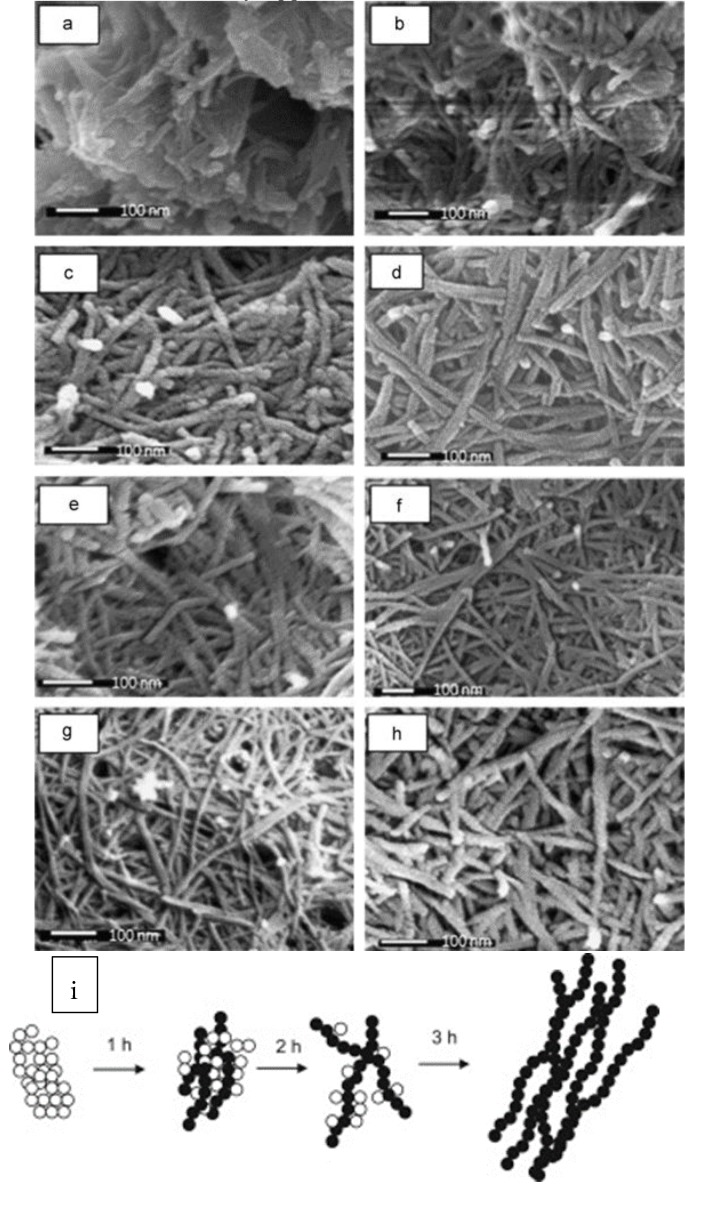
Large-scale hydrothermal synthesis is explored to produce surfactant-free seed mediated mesoporous TiO_2_ nanowires by Asiah et al. (**a**–**h**) FESEM micrographs of TiO_2_ nanowires at different growth time, (**a**) 1 h, (**b**) 2 h, (**c**) 3 h, (**d**) 4 h, (**e**) 5 h, (**f**) 6 h, (**g**) 8 h and (**h**) 10 h, respectively. (**i**) Schematic diagrams of growth evolution of TiO_2_ nanowires in time. The effect of hydrothermal growth time on the evolution of the morphology and structural properties of mesoporous TiO_2_ nanowires is shown. The initial morphology of as-prepared TiO_2_ reacted for 1 h (**a**) consists of unreacted NPs which are agglomerated and small yield of the formed nanowires with very low aspect-ratio. After 2 h (**b**) the structure of nanowires is clearly formed. However, the NPs are completely converted into nanowires after 3 h (**c**). Reproduced with permission from [65]. Copyright © 2018 Elsevier.

TiO_2_ nanowires can be also successfully obtained by using hydrothermal method, resulting in a fine shape control, as proposed by Asiah et al. that investigated a low-cost, high-purity shape-controlled synthetic strategy for the large-scale production of mesoporous TiO_2_ nanowires [65]. Titanium (IV) oxide nanopowder is used as Ti precursor solution and treated with NaOH aqueous solution. The treatment is performed by following a hydrothermal route in autoclave in a Teflon beaker at 150 °C for a reaction time ranging from 1 to 10 h. The obtained white precipitate is washed with HCl and deionized water until pH = 7 is reached. The final product, dried at 40 °C overnight and annealed at 500 °C, is found to consist of few microns long nanowires with diameters from 15 to 35 nm, according to the duration of the treatment (Figure 5). Interestingly, the hydrothermal method has also been shown to be powerful for synthesizing a three-dimensional (3D) TiO_2_ mesoporous superstructure [66,67]. TiO_2_ based superstructures with a branched architecture present an enhanced specific surface area, improved charge separation and transfer within the TiO_2_ branches, thus increasing the e^−^/h^+^ pairs lifetime and, therefore, the ROS generation [68]. 

Baloyi et al. have proposed the hydrothermal synthesis of dandelion-like structures from TiCl_4_ and water via a simple hydrothermal [68] synthesis realized in a reaction flask by adding drop-wise TiCl_4_ to the super-cooled high purity water, by using a separator funnel under vigorous stir and heating at 100 °C for 24 h. Successively, the suspension is centrifuged and washed with water to remove any chloride ions from the solid TiO_2_ and the resulting solid is dried for 16 h overnight at 120 °C. It has been proposed the obtained nanostructures grow according to a four-step reaction mechanism: (i) nucleation and NP formation; (ii) formation of spheres; (iii) further growth; and (iv) formation of flower-like TiO_2_ structures by agglomeration of the dandelions. 

**Figure 6:**
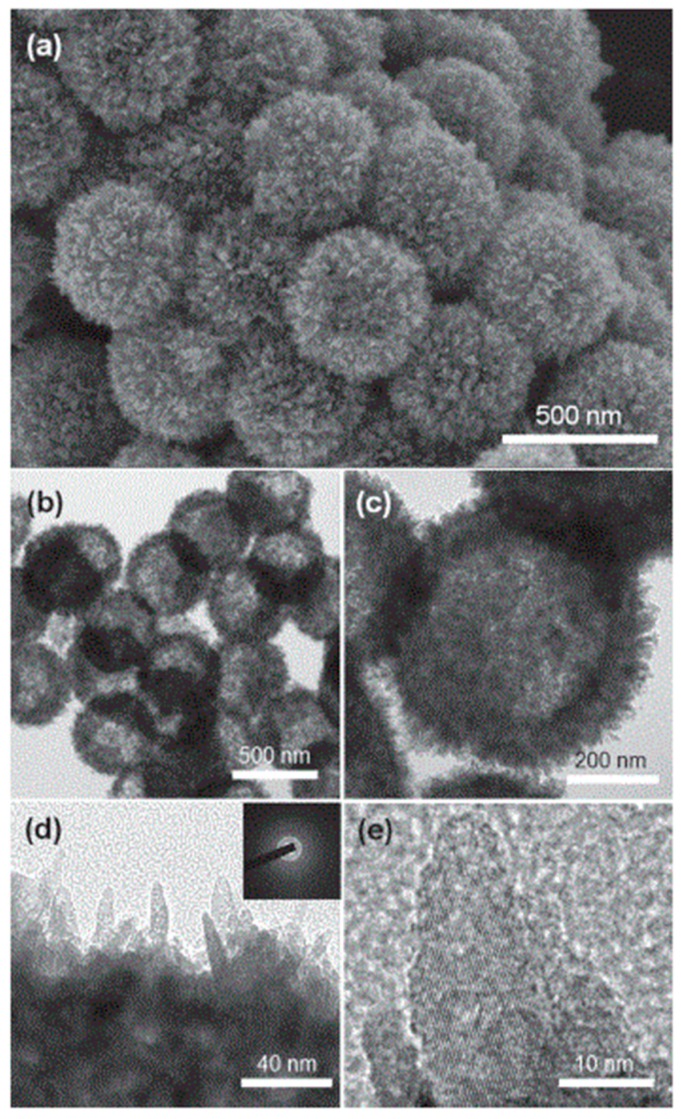
Large-scale synthesis of urchin-like mesoporous TiO_2_ hollow spheres (UMTHS) by low-temperature hydrothermal method. SEM (**a**) and TEM (**b**–**e**) micrographs of UMTHS obtained by calcining the powder after hydrothermal reaction. SEM micrographs shows that the UMTHS are monodisperse with uniform particle size and consist of radially arranged anatase nanothorns, assembling an urchin-like shaped hierarchical structure. Furthermore, TEM analysis (**b**,**c**) indicates that the spherical shell consisting of radial nanothorns. The single hallow sphere is polycrystalline due to radial orientation of nanothorns, as revealed by selected-area electron diffraction (inset of **d**). Moreover, the high-resolution TEM (**e**) shows that each nanothorn presents a single-crystal nature and possesses the lattice fringes of anatase (101) plane with a d -spacing of 0.35 nm aligned over the single nanothorn. Reproduced with permission from [67]. Copyright © 2018 John Wiley and Sons.

Pan et al. have reported the large–scale synthesis of uniform urchin-like mesoporous TiO_2_ hollow spheres (UMTHS) by a hydrothermal method based on targeted etching of self-organized amorphous hydrous TiO_2_ solid spheres (AHTSSs) (Figure 6) [67]. The growth of the UMTHSs under hydrothermal conditions has been proposed to start from the spontaneous reconstruction of surface–fluorinated AHTSSs in the presence of surface coating of polyvinylpyrrolidone (PVP). Briefly, the previously synthesized AHTSSs are treated with NaF, used as etching agent and successively with PVP. After an hour of stirring, the suspension is transferred to a Teflon-lined autoclave and kept at 110 °C for 4 h. The UMTHSs are obtained by collecting, washing with diluted NaOH solution and water, and finally calcining at 350 °C for 2 h. 

The inner structures of the materials have been demonstrated to be tunable from the conventional solid spheres to hollow and complex core-shell and yolk-shell configuration, by varying the experimental parameters and suitably protecting the inner structure by prefilling AHTSSs with PEG. The synthesized structures show a large surface area up to 128.6 m^2^/g and excellent photocatalytic performances for environmental application [67].

**Figure 7:**
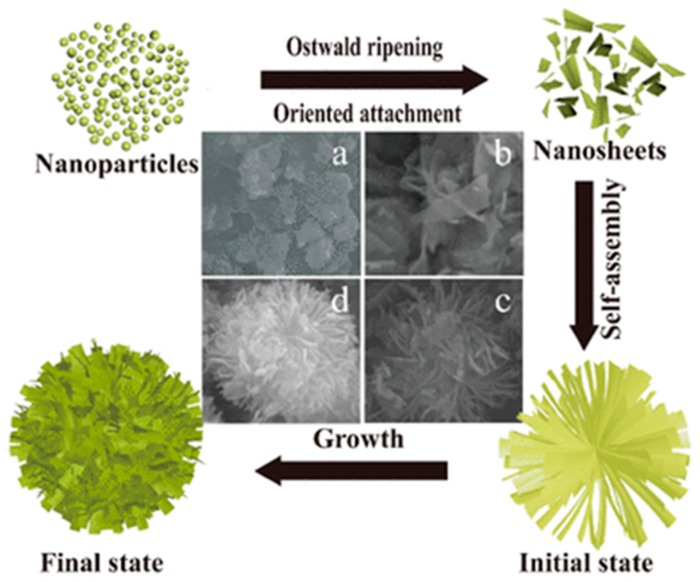
Schematic illustration of the growth evolution mechanism for 3D flower-like TiO_2_ microspheres self-assembled by nanoplates using the hydrothermal method at different time: (**a**) 3 h; (**b**) 6 h; (**c**) 9 h; (**d**) 12 h. TiO_2_ NPs after the nucleation begins to grow in the direction of orientation, forming nanosheets after dehydration. With the increasing of hydrothermal time (at 400 °C), the nanosheets are self-assembled to form a 3D flower-like structure as final state. Reproduced with permission from [69]. Copyright © 2018 Springer Nature.

Recently, Hu et al. have employed one-step hydrothermal method to synthetized 3D flower-like TiO_2_ microspheres using Ti(OBu)_4_ as titanium source and glacial acetic acid (HAc) as solvent and capping agent, at the same time [69]. Briefly, a solution of Ti(OBu)_4_ and HAc is prepared and, after stirring, is transferred into a Teflon-lined stainless-steel autoclave and heated at 140 °C for a defined period. The resulting product is collected upon centrifugation, washed with ethanol and deionized water repeatedly, dried at 60 °C for 12 h, and annealed in air. The 3D flower-like structures are formed due to the oriented assembly of nanosheets as reported in Figure 7.

TiO_2_ based photocatalyst with wormhole-like disordered structure was hydrothermally prepared by using titanium sulfate (Ti(SO_4_)_2_) as precursor of TiO_2_, and cetyltrimethyl ammonium bromide (CTAB) as a structure-directing agent [70]. The resulting photocatalyst is characterized by a narrow pore size distribution and a very high surface area of 161.2 m^2^/g, presenting an excellent adsorption ability for the removal of methyl orange (MO) and Cr(VI) from wastewater. The Ti(SO_4_)_2_ has been used as TiO_2_ precursor also for the hydrothermal synthesis of hollow TiO_2_ microspheres composed of 60 nm sized nanospheres thus resulting in a large hierarchical nanostructure characterized by a surface area and pore volume of 123 m^2^/g and 0.19 cm^3^/g, respectively [71]. The main characteristics of the synthetic approaches reported in this section have been summarized in Table 1.

## 3. Environmental Applications of Mesoporous TiO_2_

### 3.1. Application for Water Treatment

Several studies have demonstrated the effectiveness of TiO_2_ assisted photocatalysis in the removal of contaminant of emerging concern including, pesticides, synthetic and natural hormones, industrial chemicals pharmaceuticals and personal care products (PPCPs). PPCPs represent a relevant example because they are continuously released into the aquatic environment as they are extensively used in human and veterinary medicine. However, they cannot be removed using conventional wastewater treatments, thus representing a potential risk to aquatic organisms and public health. Despite mesoporous TiO_2_ is considered extremely promising for photocatalytic treatment of water, the investigations of its photocatalytic properties have been performed mainly on model molecules as organic dyes. The present section, instead, intends to report on the photocatalytic studies, currently presented in literature, on the application of mesoporous TiO_2_ for the photocatalytic gradation of molecules of environmental concern. 

The photocatalytic activity of 25–30 nm mesoporous TiO_2_ NPs with a surface area up to 40.10 m^2^/g, prepared by the sol-gel protocol described in [38] has been investigated against degradation of salicylic acid, caffeine and phenol. Phenol and its derivatives are major water pollutants in the chemical, textile, petrochemical, and paint industries. They are carcinogenic, mutagenic, and phenol derivatives can jeopardize mammalian and aquatic life [72]. Wastewaters coming from the cosmetic, paper mill, human, veterinary drugs and pharmaceutical industries, often contain salicylic acid and caffeine, which is a hazardous substance for human health [73,74]. The photocatalytic experiments have been performed using the mesoporous TiO_2_ in water suspension and uner UV irradiation with encouraging results in terms of degradation performance. Indeed, after 4h of UV light irradiation, a degradation efficiency of 92% and 59% has been achieved for phenol and caffeine respectively, while for the salicylic acid a degradation amount of 88% has been reached after 3 h of reaction [38].

**Figure 8:**
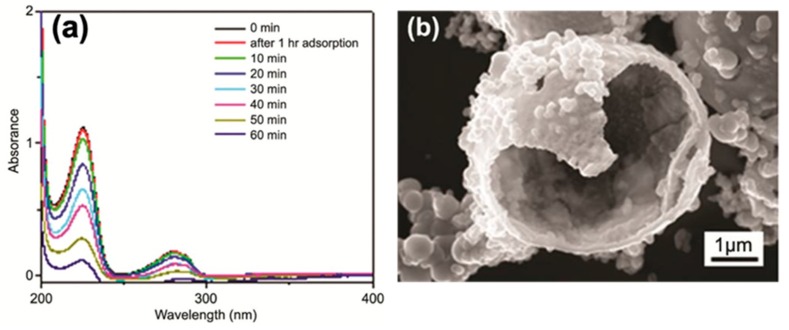
(**a**) Ultraviolet (UV)–visible absorption spectra of the 4-CP containing sample, in the course of the heterogeneous photocatalytic experiment assisted by hollow TiO_2_ microspheres as photocatalyst. The TiO_2_ has been separated from the aqueous solution of 4-CP by filtration, before recording the absorption spectrum (**b**) FESEM high magnifications micrography of TiO_2_ hollow microspheres. Reproduced with permission from ref. [75] http://creativecommons.org/licenses/by-nc-nd/2.5/in/.

Hollow of TiO_2_ microspheres have demonstrated to be effective in the photocatalytic degradation of 4-chlorophenol (4-CP), promoting the 90% photodegradation of 4-CP within 1 h under UV light irradiation (Figure 8). The potential reusability of TiO_2_ hollow microspheres, has been investigated by performing three cycles of reuse and any significant change of the photocatalytic performance has been detected. Also, no relevant alteration in terms of the 4-CP degradation rate constant has been measured, thus highlighting the effectiveness of this material for photocatalytic water treatment [75]. 

### 3.2. Air Treatment

Photocatalytic processes, assisted by TiO_2_, have in recent years shown a great potential in the abatement air pollutants as NO_x_ gases (commonly referred to as nitrogen monoxide, NO, and nitrogen dioxide NO_2_), and volatile organic compounds (VOCs) [76]. VOCs represent a major group of indoor air pollutants responsible of the production of tropospheric ozone and secondary organic aerosol with several adverse health effects [77]. Indeed, several VOCs such as halogenated hydrocarbons, ketones, alcohols and aromatic compounds are toxic and carcinogenic pollutants. Moreover, the interaction of NO_x_ with VOCs provokes the formation of by-products even more dangerous than NO_x_ gases, such as nitrous acid and peroxyacyl nitrate (PAN) [3]. Recently, several examples concerning the application of mesoporous TiO_2_ material to removal of NO_x_ and VOCs have been reported in literature [78,79]. 

#### 3.2.1. Photocatalytic Abatement of NO_x_ by Using Mesoporous TiO_2_

**Figure 9:**
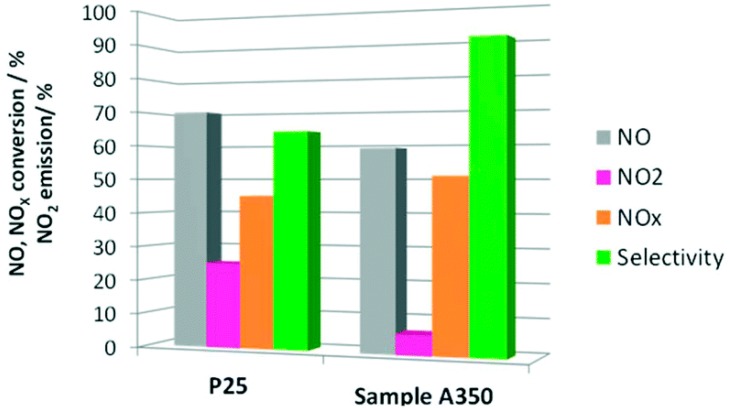
Mesocrystalline TiO_2_ NPs are synthesized using a hydrothermal method as a photocatalyst for NOx abatement. The diagram reports NO conversion (%, grey), NO_2_ released (%, pink), NOx conversion (%, orange) and selectivity values (%, green) for A350 Titania (“as prepared” sample) and P25 TiO_2_ (as reference material) after five hours of light irradiation. The selectivity is here intended as the complete conversion of NO in nitrate or nitric acid, that leads to the efficient removal of NOx species. In terms of NOx removal capability, the prepared titania appears to be a desirable photocatalyst, which combines high efficiency in photochemical NO conversion (grey bar) and selectivity (green bar), resulting in the highest NOx conversion (orange bar). Reproduced with permission from [81]. Copyright © 2019.

Mesoporous TiO_2_-based films are widely studied for photodecomposition processes based on the surface-adsorbed reactants, because the large surface area of mesoporous TiO_2_ determines an increased amount of adsorbed resulting in an enhancement of their decomposition rate [79,80]. A relevant example of mesoporous TiO_2_-based film applied to air purification has been reported by Kalousek et al. The TiO_2_-based film is synthetized by a template-assisted method based on the evaporation-induced self-assembly mechanism which allows to control the film thickness and consequently its surface area and pore volume [79]. NO_x_ degradation assisted by porous films has been investigated and compared with that achieved by means of commercial Pilkington Active Glass. The film obtained exhibits a very high photocatalytic efficiency probably due to their peculiar morphological properties such as large surface area and pore volume. Such a valuable photoactivity has been assumed to arise from the local increase of compound’s partial pressure reacting in the nanopores or cavities of the film in proximity to the photocatalytic sites. Moreover, the high selectivity towards nitric acid has been ascribed to the strong adsorption of the intermediate products. Balbuena et al. have reported as innovative solution for environmental remediation, mesocrystalline anatase NPs synthesized by hydrothermal method easily up scaled to industrial level [81]. The obtained TiO_2_ photocatalyst (namely TiO_2_ A350) exhibits a suitable nanostructure and porosity, with a surface area and pore volume of 63.5 m^2^/g and 0.22 cm^3^/g, respectively. The oxidation of NO is evaluated by using a laminar flow reactor and an artificial sunlight as irradiation (25 and 550 W m^−2^ for UV and visible irradiances, respectively). The final product presents a higher degradation efficiency and selectivity for the NO_x_ abatement than TiO_2_ P25 (Figure 9), due to probably to the presence of mesopores which increases the surface area of the samples and the accessibility for the reactant molecules.

#### 3.2.2. Photocatalytic Abatement of Volatile Organic Compounds (VOCs) by Mesoporous TiO_2_

**Figure 10:**
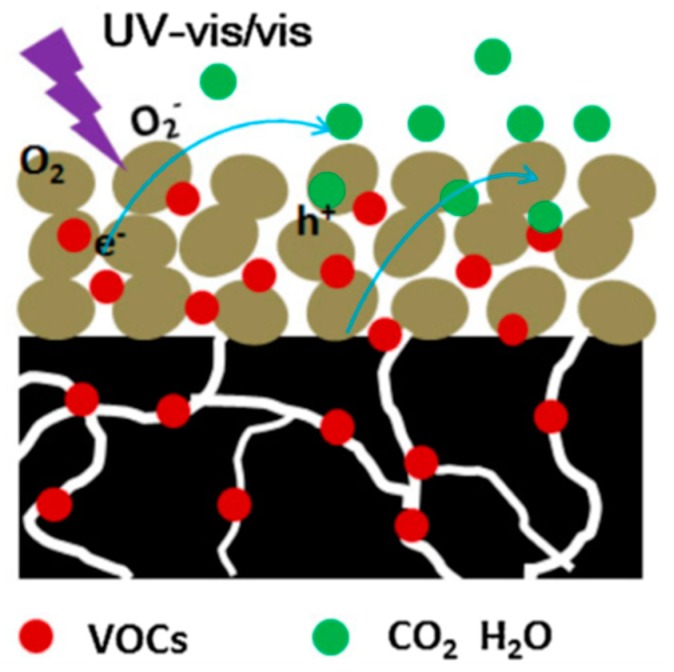
Schematic mechanism of the proposed photocatalytic oxidation of volatile organic compounds (VOCs) in O_2_ under UV–vis and visible light irradiation for mesoporous TiO_2_/hydro-CF. Mesoporous TiO_2_ films (brown spots) can be excited to form e^−^/h^+^ under UV–vis and visible light irradiation. The h^+^ is one of the strong oxidant for gaseous VOCs mineralization. Moreover, TiO_2_/hydro-CF substrate adsorb the VOCs molecules increasing the concentration of VOCs at the interfacial between of photocatalyst and substrate, as well as facilitate the mass transfer of VOCs due to the macroporous structure. Reproduced with permission from [82]. Copyright © 4472401418350, 2018 Elsevier.

Similarly, in NO_x_ degradation, photocatalytic processes assisted by TiO_2_ surfaces may potentially remove VOCs, because photogenerated ^•^OH radicals and superoxide radicals can promote their mineralization leading to water vapour H_2_O and carbon dioxide (CO_2_) [15,82]. Generally, the degradation of VOCs leaves a recalcitrant carbonaceous residue accumulation on the photocatalyst surface as a result of incomplete oxidation of VOCs, which causes catalyst deactivation decreasing the photocatalytic efficiency [83]. Several efforts have been devoted to remove this drawback that, in spite of the long-term photocatalytic stability, still represents a big challenge for the degradation process of VOCs. A possible solution is given by the use of mesoporous TiO_2_, that, due to the channels in the structure, increases the density of highly accessible active sites thus promoting the diffusion of reactants and products as a consequence of the capability of absorbing pollutant molecules [83]. A mesoporous TiO_2_-based system has been fabricated by Ji et al., as a promising photocatalyst for the oxidation of gaseous benzene [83]. This material exhibits a higher photocatalytic performance and stability towards benzene degradation compared to commercially available TiO_2_ P25. In particular, among others, the material obtained upon calcination at 400 °C has shown a good mesoporous structure and superior capacity for benzene adsorption. Nowadays, photocatalytic materials for filter represent another relevant application devoted to air purification systems [84]. In order to fabricate effective photocatalytic filters porous foams have been identified as solid supports due to their high open porous structure with large surface area, that provide excellent structure for gas passing while maintaining a high level of surface contact and low level of pressure drop. Among the photocatalytic filters for commercial environmental purifiers, TiO_2_-immobilized ceramic foams represent a very effective system, especially for reducing indoor air pollution caused by VOCs [84]. Recently, Qian et al. have fabricated hydro-carbon foams (CFs) as support for TiO_2_ photocatalyst for an indoor air treatment application resulting in TiO_2_/hydro-CFs [82]. These CFs are prepared by waste polyurethane foams (PUF), while phenolic resin is used as a hard template and a carbon source. Mesoporous TiO_2_ coatings are uniformly deposited on the foam by a self-assembly sol-gel method. The TiO_2_/hydro-CF exhibit obtained enhanced photocatalytic oxidation activity for VOCs under UV–vis and visible light irradiation, probably due to the synergic combination of TiO_2_ and CF that promote the mass transfer and the accumulation of pollutant molecules at the interface photocatalyst/substrate according to the mechanism proposed in Figure 10. 

## 4. Conclusions

Here an overview of recently reported synthesis protocols suited for the large- scale preparation of mesoporous TiO_2_ has been provided. The reviewed syntheses have been selected to comply with the criteria of scalability, morphological and structural control, and use of safe and affordable TiO_2_ precursors. Mesoporous TiO_2_ as a photocatalyst offers multifold advantages such as high specific surface area, the high density of surface hydroxyl moieties, and improved absorption of UV. Several examples of TiO_2_ mesoporous TiO_2_ have been accounted for and discussed. Overall mesoporous TiO_2_ has been demonstrated to show a great potential in the field of TiO_2_-based environmental photocatalysis, also considering that currently available synthetic routes are suitable for up-scaling production of TiO_2_ NPs, while still ensuring a high control over size, shape, structure and textural properties. However, great efforts are still required in order to fully elucidate the interaction with UV light, accomplish useful surface functionalization and thoroughly assess the safety issues related to the real application of nanosized TiO_2_-based photocatalyst.

## Figures and Tables

**Table 1:** Main characteristics of the reported synthetic approaches for the preparation of mesoporous TiO_2_.

Synthetic Routes	Control Parameters	Particle Size	Pore Size	Specific Surface Area	Main Advantages	Main Drawbacks
**Sol-gel methods** [33,34,35,36,37,38,39]	pH, calcination temperature	Micrometer and sub-micrometer aggregates of nanoparticles	Pore volume from 0.18 cm^3^/g to 0.50 cm^3^/g; Pore size from 9.16 nm to 16.9 nm	From 70 m^2^/g to 150 m^2^/g	Low cost; User-friendly protocol Special facilities not required	Difficult control on the morphological and textural properties; calcination step required
**Synthesis in room temperature ionic liquids** [41,42,43,44,45,46]	Viscosity, temperature, stirring	Crystallite size 3–6 nm	Pore size 5 nm to 8 nm	554 m^2^/g	Low temperature required	High cost due to the use of ionic liquid
**Hydrothermal synthesis** [47,48,49,50,51,52,53,54,55,56,57,58,59,60,61,62,63,64,65,66,67,68,69,70,71]	pH, temperature, pressure, filling volume, hydrothermal treatment duration	Micrometric particles formed by nanoparticles from 3.4 nm to 27 nm	Pore volume from 0.18 cm^3^/g to 0.50 cm^3^/g Pore size from 3.4 nm to 27 nm	From 25 m^2^/g to 395 m^2^/g	High control over morphology and textural properties; Use of aqueous suspensions; Calcination step not always required: Facile photocatalyst recovering	Use of a specific facility (autoclave)
